# Fibro-osseous Pseudotumor of the Toe

**Published:** 2020-02-22

**Authors:** Daisuke Atomura, Takaya Makiguchi, Takahiro Yamaguchi, Hideharu Nakamura, Yukie Yamatsu, Ken Shirabe, Satoshi Yokoo

**Affiliations:** ^a^Department of Oral and Maxillofacial Surgery and Plastic Surgery; ^b^Department of General Surgical Science, Gunma University Graduate School of Medicine, Maebashi, Gunma, Japan

**Keywords:** fibro-osseous pseudotumor, myositis ossificans, bizarre parosteal osteochondromatous proliferation, osteosarcoma, pyogenic granuloma

## CASE DESCRIPTION

**Figure 1:**
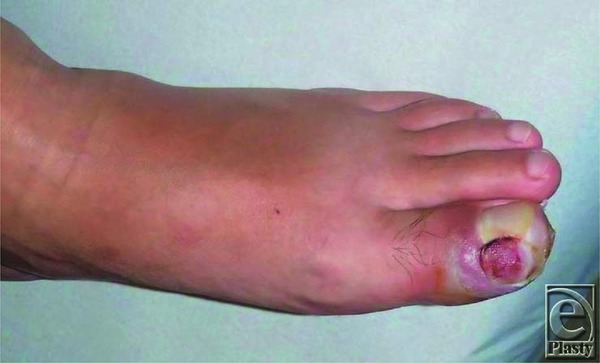
Reddish and ulcerated lesion in the left great toe.

**Figure 2:**
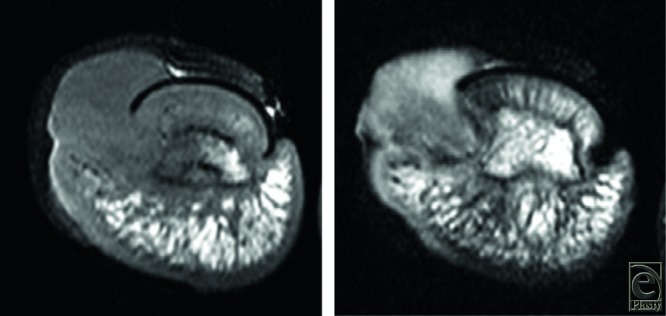
Magnetic resonance imaging revealed a mass occupying the medial aspect of the distal phalanx, with low signal intensity on T1-weighted images and intermediate to high signal intensity on T2-weighted images.

**Figure 3:**
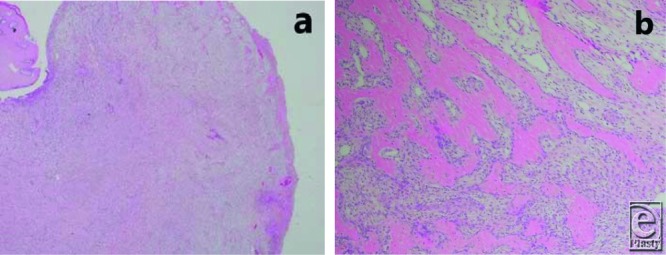
Histopathological findings. (*a*) Low-magnification view showing the architecture with ulcerated epidermis. (*b*) High-magnification view showing myofibroblastic and fibrovascular proliferations, with mature woven bone rimmed by osteoblasts. No evidence of abnormal mitotic activity or cellular atypia was observed.

**Figure 4:**
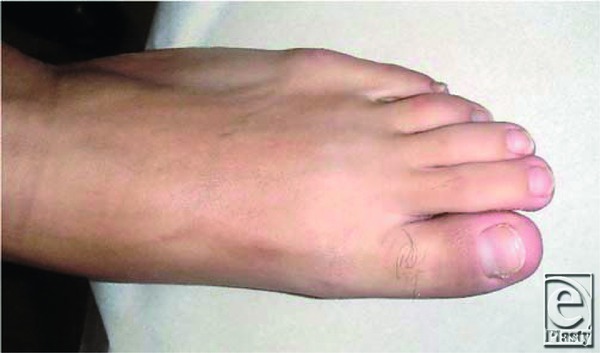
Follow-up image showing no recurrence of the mass after 6 months.

A 40-year-old man presented with a progressively enlarging mass in the left great toe for a few months. The patient was diagnosed with pyogenic granuloma and was treated with cryotherapy several times. However, regression was not observed. Clinical examination revealed a 1.5 × 1.0-cm reddish nodule on the medial side of the toe (Fig 1). Radiological evaluation revealed a soft-tissue mass without calcification, and magnetic resonance imaging (MRI) revealed a mass adjacent to the distal phalanx (Fig 2). Excisional biopsy was performed, with the patient under local anesthesia. The mass was not connected to the bone and was easily dissected from the surrounding tissues. Histopathological examination revealed that the lesion comprised myofibroblastic and fibrovascular proliferations. Mature bony trabeculae rimmed by osteoblasts were observed in the peripheral area, which was diagnosed as a fibro-osseous pseudotumor (FOP) (Figs 3*a* and 3*b*). Clinical recurrence was not observed after 6 months (Fig 4).

## QUESTIONS

1. What is an FOP?
2. How is an FOP diagnosed?
3. What should be considered for the differential diagnosis of an FOP?
4. How can an FOP be treated?

## DISCUSSION

Dupree and Enzinger^1^ first described FOP as a benign reactive fibroblastic proliferation with osseous lesion. The lesion was previously referred to as parosteal fasciitis and florid reactive periostitis and later defined as a lesion originating from subcutaneous tissues without muscular involvement. FOP occurs in young to middle-aged adults, and men are more commonly affected than women. The lesion is usually asymptomatic, but patients may often complain of pain and erythema. The etiology of FOP is unclear. However, it usually occurs after a trauma. That is, a history of trauma is observed in approximately 40% of cases.^1^ The sites commonly involved are the proximal phalanx, metacarpal, and metatarsal regions.^2^ The lesion is more likely to be observed in the subcutaneous tissues of the hand, and the toe is not a common site. In fact, to the best of our knowledge, only 17 cases have been reported in the literature.^1^^-^5

Combined radiology and MRI and most importantly histological findings are used for the diagnosis of FOP. The radiological characteristics of the lesion are soft-tissue swelling located adjacent to the cortex, without any calcification and rarely periosteal reaction. The MRI characteristics are subcutaneous, well-circumscribed, soft-tissue mass, with low to intermediate signal intensity on T1-weighted images, variable signal intensities on T2-weighted images, and lack of continuity to the bone marrow.^6^ Histopathological examination is the most important examination for diagnosis because it reveals a well-delineated, predominantly dermal spindle cell lesion comprising mature and immature fibroblastic foci associated with osteoid formation.^2^ Cellular atypia and mitotic activity are not frequently observed but may be identified during the growth phase. As the lesion matures fully, a zoning phenomenon is noted, which is a characteristic of myositis ossificans (MO), and fibroblast proliferation surrounded by a myxoid matrix is observed at the center of the lesion, with osseous trabeculae rimmed by osteoblasts in the peripheral area.^5^

The main differential diagnoses of FOP are MO, bizarre parosteal osteochondromatous proliferation (BPOP), and osteosarcoma. FOP is the most similar to MO, and recently, FOP was considered a subtype of MO. Both FOP and MO have similar histopathological features, such as fibroblastic proliferation and intramembranous ossification. However, FOP occurs in the more distal extremity, and a superficial lesion without zonation pattern is observed. Meanwhile, MO develops in the deeper aspect of the proximal soft tissues, with a zonation pattern. BPOP may clinically simulate FOP; BPOP and FOP are similar in terms of their histological and radiological characteristics, and they occur in the soft tissues of superficial lesions such as the digits. Moosavi et al^7^ described attachment to the bone, with endochondral ossification in BPOP but not in FOP, which is an extraosseous lesion. Although mitotic activity and cellular atypia were not observed in our patient, such characteristics may be observed in FOP and the lesion should not be confused with malignancy, such as osteosarcoma, which presents with destructive stromal invasion and obvious cytologic atypia. FOP mainly occurs in young adults and osteosarcoma in older individuals.

Complete excision is the gold standard treatment of FOP, with a low recurrence rate and excellent prognosis when the lesion is completely resected. Extended resection should be avoided because FOP is benign, and malignant transformation into osteosarcoma is extremely rare. However, several studies have reported unnecessary amputation that was performed because of misdiagnosis.^7^^,^^8^ Malignancy must be ruled out, and excision of normal tissue margins must be performed to prevent unnecessary amputation and preserve function.^8^ Thus, excisional biopsy and histopathological examination are recommended to diagnose FOP accurately, which will subsequently lead to timely and appropriate treatment.

## SUMMARY

FOP is a rare and benign ossifying soft-tissue tumor affecting the subcutaneous tissues of the hands and less frequently of the toes. Osteosarcoma is the most significant differential diagnosis, and the treatment of the lesion is complete surgical excision. Excisional biopsy and histopathological diagnosis are important for proper treatment.

## References

[1] Dupree WB, Enzinger FM (1986). Fibro-osseous pseudotumor of the digits. Cancer.

[2] Chaudhry IH, Kazakov DV, Michal M, Mentzel T, Luzar B, Calonje E (2010). Fibro-osseous pseudotumor of the digit: a clinicopathological study of 17 cases. J Cutan Pathol.

[3] Meani RE, Bloom RJ, Battye S, Chamberlain AJ (2016). Subungual fibro-osseous pseudotumour of the toe. Australas J Dermatol.

[4] Chan KW, Khoo US, Ho CM (1993). Fibro-osseous pseudotumor of the digits: report of a case with immunohistochemical and ultrastructural studies. Pathology.

[5] Sleater J, Mullins D, Chun K, Hendricks J (1996). Fibro-osseous pseudotumor of the digit: a comparison to myositis ossificans by light microscopy and immunohistochemical methods. J Cutan Pathol.

[6] Jawadi T, AlShomer F, Al-Motairi M, Al-Qahtani A, Alfowzan M, Almeshal O (2018). Fibro-osseous pseudotumor of the digit: case report and surgical experience with extensive digital lesion abutting on neurovascular bundles. Ann Med Surg.

[7] Moosavi CA, Al-Nahar LA, Murphey MD, Fanburg-Smith JC (2008). Fibroosseous pseudotumor of the digit: a clinicopathologic study of 43 new cases. Ann Diagn Pathol.

[8] Coleman RA (2005). Fibro-osseous pseudotumour of the digit—amputation for a benign but aggressive lesion. J Hand Surg Br.

